# Interaction of Self-Regulation and Contextual Effects on Pre-attentive Auditory Processing: A Combined EEG/ECG Study

**DOI:** 10.3389/fnins.2019.00638

**Published:** 2019-06-19

**Authors:** Yu Hao, Lin Yao, Qiuyan Sun, Disha Gupta

**Affiliations:** ^1^Department of Design and Environmental Analysis, Cornell University, Ithaca, NY, United States; ^2^School of Electrical and Computer Engineering, Cornell University, Ithaca, NY, United States; ^3^Department of Nutritional Science, Cornell University, Ithaca, NY, United States; ^4^School of Medicine, New York University, New York, NY, United States

**Keywords:** contextual effect, regulation, adaptation, sensitivity, attention, pre-attentive, mismatch negativity (MMN)

## Abstract

Environmental changes are not always within the focus of our attention, and sensitive reactions (i.e., quicker and stronger responses) can be essential for an organism's survival and adaptation. Here we report that neurophysiological responses to sound changes that are not in the focus of attention are related to both ambient acoustic contexts and regulation ability. We assessed electroencephalograph (EEG) mismatch negativity (MMN) latency and amplitude in response to sound changes in two contexts: ascending and descending pitch sequences while participants were instructed to attend to muted videos. Prolonged latency and increased amplitude of MMN at fronto-central region occurred in ascending pitch sequences relative to descending sequences. We also assessed how regulation related to the contextual effects on MMN. Reactions to changes in the ascending sequence were observed with the attention control (frontal EEG theta/beta ratio) indicating speed of reaction, and the autonomous regulation (heart-rate variability) indicating intensity of reaction. Moreover, sound changes in the ascending context were associated with more activation of anterior cingulate cortex and insula, suggesting arousal effects and regulation processes. These findings suggest that the relation between speed and intensity is not fixed and may be modified by contexts and self-regulation ability. Specifically, cortical and cardiovascular indicators of self-regulation may specify different aspects of response sensitivity in terms of speed and intensity.

## 1. Introduction

With the dynamic nature of the ambient environment, organisms as ensembles of both brain and body interact with the environment while maintaining equilibrium through adaptation and regulation (Helson, 1964; Damasio, 1994). Although it is generally accepted that this adaptation results from the integration of brain, body, and environment (Helson, 1964), the neural basis in this system is less well-understood. One role that the brain plays is the modulation of perceptual sensitivity based on contextual information from the environment. For example, perception of a tone pitch was biased toward previously heard pitches, reflecting the temporal binding of successive frequency components (Chambers et al., 2017). Unattended changes in a regular context were associated with higher neural responses than in a random context, indicating that the brain was more sensitive to change in a predictable context (Southwell and Chait, 2018). This responsiveness to environmental changes is essential for survival, especially when changes are not always in the focus of attention. However, it remains unclear how the brain responds to changes in unattended stimuli in different dynamic environmental contexts and in what way the brain and body work together to regulate response sensitivity to such stimuli. Sensitivity to stimuli has usually been shown to be reflected in a quicker and stronger neural response. Thus, the purpose of this study is to examine how the perceptual sensitivity (i.e., speed and intensity) of the brain's responses to unattended changes modulated by the environmental contextual information and its association with regulation mechanisms from brain and body.

We study unattended sensory information processing by measuring neural responses to ambient acoustic stimuli, following the well-established electroencephalograph (EEG) based Mismatch Negativity (MMN). MMN is evoked by a deviant event in a sequence of repeated or familiar events (the standards) when people are not focusing on the stimuli (Näätänen et al., 2011). When immersed in ambient environmental stimuli, the brain predicts sensory inputs and compares the incoming stimuli with top-down predictions, eliciting an MMN when a prediction error is detected (Friston, 2005; Garrido et al., 2009). Our experiment used ascending pitch sequences or descending pitch sequences as contexts. The primary aim was to assess the effect of context on the MMN response in terms of intensity (i.e., amplitude) and speed (i.e., latency, the timing from stimulus onset to response peak).

Prior work indicates that the pitch of environmental sounds alters perceived valence and arousal of auditory stimuli (Juslin and Laukka, 2003; Ilie and Thompson, 2006; Ma and Thompson, 2015). The stimuli were designed to have equal intensity, but the higher pitch stimuli would also be perceived as louder and the lower pitch as softer, as per loudness-frequency curves. The pitch and loudness are fundamental characteristics of speech that embody emotional context (Scherer and Oshinsky, 1977). The regular pattern of the ambient sound context might generate an environmentally induced affective state which triggers self-regulation processes. Thus, we hypothesized that arousal and biological regulation mediate contextual effect on response to unattended changes. Usually, MMN latency and amplitude have been found to be related, with a quicker (smaller latency) and a larger (greater amplitude) MMN response indicative of sensitivity to change, both because of variations in deviant stimuli and cognitive decline (Näätänen and Kujala, 2011; Näätänen and Kreegipuu, 2012; Näätänen et al., 2012; Schirmer et al., 2016). Therefore, the second aim was to understand MMN response intensity and speed in relation to arousal and regulation processes. Self-regulation involves volitional and non-volitional components enabled by executive functioning and autonomous system (Blair, 2010; Hofmann et al., 2012). As a biomarker of executive functioning, EEG frontal theta/beta ratio reflects prefrontal cortex-mediated executive control over attentional and emotional information, with lower frontal theta/beta ratio representing better focused attention and emotion regulation (Angelidis et al., 2018). As a biomarker of autonomic regulation, heart rate variability (HRV) reflects stress and regulated emotional response, with higher values representing better regulation (Thayer et al., 2012). Therefore, we applied these biomarkers to examine MMN responses in different contexts.

## 2. Materials and Methods

### 2.1. Participants

Twenty right-handed students (*M* = 20.38y, *SD* = 2.64; 14 females) from Cornell University participated in this study. Exclusion criteria were the use of any medication that could affect nervous and cardiovascular systems and any history of neurological disorders. The study was approved by the Cornell University Institutional Review Board. Informed consent was obtained, and participants were compensated with either class credits or $20.

### 2.2. Experiment

**Figure 1:**
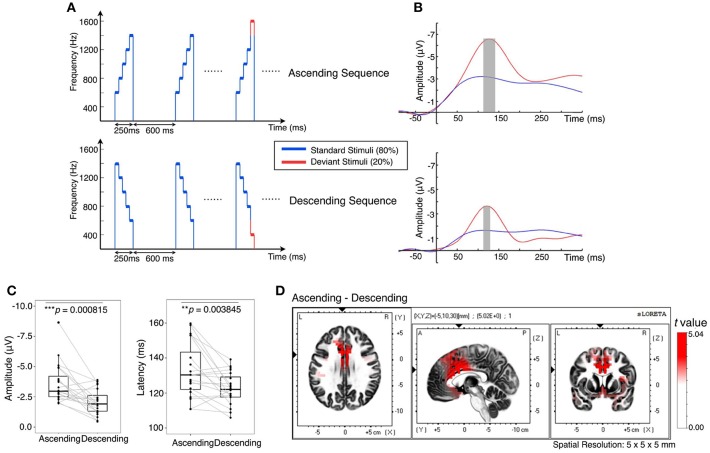
Contextual effect on brain responses to unattended sound changes. **(A)** Twenty percent of the stimuli set in either context had a deviant tone (plotted in red): a 200 Hz additional pitch increment or decrement tone in substitution to the fifth tone of the standard stimuli. **(B)** Grand average MMN at electrodes having the largest difference between standard and deviant in the fronto-central region. Gray bars are peak intervals (peak latency ± *SD, N* = 20) for ascending context at 118–149 ms and descending context at 114–132 ms. **(C)** MMN amplitude was greater in the ascending context (–3.60 ± 1.65 μV) than in the descending context (–2.08 ± 1.06 μV), *t*_(19)_ = –3.97, *p* = 0.0008. MMN latency was greater in the ascending context (132.84 ±15.53 ms) than in the descending context (122.55 ± 9.15 ms), *t*_(19)_ = 3.29, *p* = 0.0038. **(D)** Significant increase (when *t* > 2.093 for corresponding voxel) in current source density of the MMN in the ascending condition compared with the descending condition at the respective peak intervals.

Participants were individually immersed in auditory oddball streams that were either rapidly ascending in pitch (600–1,400 Hz) or descending in pitch (1,400–600 Hz), while they were asked to watch a self-selected silent documentary. The deviant in each stream was the last pitch, 1,600 Hz instead of 1,400 Hz in an ascending stream and 400 Hz instead of 600 Hz in a descending stream (Figure 1A). We did not compare the difference between low and high ends but addressing the low- and high-end changes with contexts of constantly changing auditory sequences. Therefore, the non-linear auditory perception of pitch on MMN will be tested in the future study.

A single sound stimulus was a five-tone cluster in ascending or descending sequence conditions. Each tone 50 ms, therefore a stimulus was 250 ms. The auditory paradigm involved the presentation of 1,260 sound stimuli as a sequence of “standard” stimuli (80%) randomly interspersed by “deviant” stimuli (20%). Each type of context (ascending or descending) was divided into 7 runs with each consisted of 144 standards and 36 deviants maintaining the 80/20 ratio. The first 10 stimuli in each run were specifically standards (to establish the expectation), followed by a pseudo-random distribution of standards and deviants, avoiding consecutive deviants. The runs of the two sequence contexts were randomly presented. EEG was recorded using 128-channel BioSemi system at a sampling rate of 512 Hz. ECG was recorded from three BioSemi electrodes placed on left and right abdominal region and the region below the right collarbone. Stimuli were presented via BCI2000 software at 55 *dBA* via speakers placed 40 cm in front of the participant. The sound pressure level was measured with a sound meter at the location of subject's head.

### 2.3. Analysis

EEG was re-referenced to the algebraic average of left and right mastoids, and was bandpass filtered between 0.1 and 55 Hz. For event related potential (ERP) analysis, the signal was further filtered within [0.5 10] Hz (for different filtering comparison see Figure S1). Bad channels were identified and spherically interpolated. Data was epoched into [–100 350] ms trials, where time 0 was defined as the time of stimulus onset asynchrony which was 200 ms after the start of the complex tone pattern, which was also the start of the last tone. The artifacts due to eye movements, muscle and cardiac activity were removed with Independent Component Analysis, using the EEGLab toolbox (Delorme and Makeig, 2004). The epochs were averaged across trials for each channel to obtain the evoked response. The [–100 0] ms period before the last tone was used for baseline correction. All the standard stimuli and also the deviant stimuli were averaged, and then the MMN waveform was calculated as the difference between ERP of the deviant stimuli and the standard stimuli. For the MMN waveform with baseline correction during the trial interval [–300 -200] ms, see Figure S2. We used the coefficient-of-determination (*r*^2^) to quantify the difference between standard and deviant responses in each time point after the stimulus. The *r*^2^ was calculated as the square of the Pearson correlation coefficient between the amplitude in each time point and the trial label (standard or deviant). Then we individually selected the fronto-central channel that had the negative peak with largest *r*^2^ within [80 200] ms (Garrido et al., 2009).

Current source densities in each voxel between two conditions (ascending vs. descending) were compared by randomization tests on paired data (5,000 permutations). Based on statistical non-parametric mapping (SnPM; for details see Holmes et al., 1996) corrected for multiple comparison, we used the sLORETA (Pascual-Marqui, 2002) software to perform “non-parametric randomization” of the data. Besides, We estimated the ratio of the theta (4–7 Hz) and beta (14–30 Hz) power at the frontal regions (Angelidis et al., 2018), including C21, C32, and C10 channels. In terms of the HRV analysis, We used the Pan-Tompkin algorithm to extract the QRS complex and detect the peak of the R waveform, and then measured the R-R interval between successive R-to-R peak. Then we used the root mean square of the successive differences (RMSSD) of the R-R interval to quantify the HRV (Stein et al., 1994). These variables were calculated for each participant under each sequence context across runs. We explored the non-linear relationship between these biomarkers using the Spearman correlation test.

Paired *t*-test was used to examine the MMN amplitude and latency between ascending and descending context. Linear mixed effect models were used to investigate the interaction of self-regulation biomarkers and contexts on MMN amplitude and latency respectively. High and low theta/beta ratio and HRV were plotted ±1 *SD* from the mean in the Figures.

## 3. Results

MMN was obtained by subtracting the response to the standard stimulus (blue) from the response to the deviant stimulus (red) (Figure 1B). The ascending context elicited a slower (*p* < 0.01) but larger (*p* < 0.001) MMN than the descending context (Figure 1C). Furthermore, current source density of MMN in the ascending context was significantly higher than descending context in the insula, *p* < 0.01, and in the cingulate gyrus including anterior cingulate cortex (ACC), *p* < 0.001 (Figure 1D). Moreover, the source density of MMN filtered within broad-band [0.5 55] Hz showed a similar result (Figure S3). These results are consistent with the error detection mechanism mentioned above as the anterior cingulate cortex contributes to attention, which serves to regulate both cognitive and emotional processing (Etkin et al., 2011). On the other hand, the insula receives viscerosensory inputs and responsible for affective experience, particularly in negative emotion suppression (Etkin and Wager, 2007; Giuliani et al., 2011). Altogether, the results indicate that the brain is more sensitive to changes in the ascending context and executed more regulation procedures.

**Figure 2:**
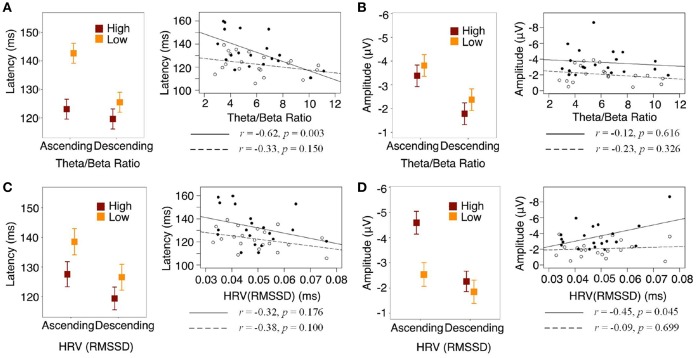
Contextual effect interacted with self-regulation. Solid lines and dots represent ascending condition, dash lines and circles represent descending condition. **(A)** Statistical interaction modeling showed that theta/beta ratio interacted with context on MMN latency, $\chi_{\left(1\right)}^{2}$ = 5.10, *p* = 0.0239. **(B)** Theta/beta ratio did not interact with context on MMN amplitude, $\chi_{\left(1\right)}^{2}$ = 0.04, *p* = 0.8448. **(C)** HRV did not interact with context on MMN latency, $\chi_{\left(1\right)}^{2}$ = 0.24, *p* = 0.6234. **(D)** HRV marginally interacted with context on MMN amplitude, $\chi_{\left(1\right)}^{2}$ = 3.67, *p* = 0.0555.

We then examined how MMN amplitude and latency were associated with regulation biomarkers. A non-linear correlation (Spearman correlation test) existed between theta/beta ratio and HRV (*p* < 0.01): moderate theta/beta ratio corresponded to the highest HRV and, as theta/beta ratio increased, HRV declined. We found that both theta/beta ratio and HRV were not significantly different between ascending and descending context (Figure S4). However, higher theta/beta ratio was associated with smaller MMN latency and higher HRV was associated with larger MMN amplitude in the ascending context, whereas no correlations were found in the descending context (Figures 2A–D).

## 4. Discussion

Sensitivity to stimuli means a quicker and/or stronger neural response. Interestingly, in our study, the ascending auditory context induced stronger yet slower neural responses, i.e., a larger but later MMN. This phenomenon is different from previous findings wherein the latency and the amplitude of MMN were negatively correlated that together reflect the sensitivity to change (Näätänen and Kujala, 2011; Näätänen and Kreegipuu, 2012; Näätänen et al., 2012; Schirmer et al., 2016). If deviants in ascending were simply more salient than in descending, they would be expected to elicit greater amplitude and smaller (or similar) latency. Thus, these data suggest (1) reaction intensity and speed may reflect different aspects of sensitivity, which were possibly processed by different mechanisms, and (2) when exposed to different contexts, these mechanisms could be differentiated. Moreover, the MMN latency and amplitude did not correlate with each other in any context or cross contexts (Figure S5), further supporting that deviant stimuli with higher pitch not only influence sensitivity but are also associated with the interaction of regulation and context. Regulation biomarkers can account for neural responses to unattended changes, but only under some specific conditions, such as the ascending pitch context described in this study. Further, the response intensity and speed can be accounted for by cortical and cardiovascular biomarkers of regulation respectively.

The anterior cingulate and insula are involved in the central autonomic neural network, which is a part of an internal regulation system wherein the brain controls visceromotor and neuroendocrine processes, essential for adapting to environmental demands (Benarroch, 1993). As during the ascending context, more activation in ACC and insula was observed, the context-related heightened arousal might trigger more regulation, so that participants could adapt. Participants with higher HRV throughout the experimental sessions elicited stronger MMN, reflecting better regulation during unexpected changes.

Attention has been shown to influence MMN generation (Näätänen et al., 1993; Snyder et al., 2012). In our study, though participants were instructed to focus their attention on documentary videos, their neural responses to deviant stimuli differed because individual attentional control as one of the regulation processes was manifested. Slower response was observed in individuals with low theta/beta ratio. These individuals were superior in attentional control during focused attention tasks (Derakshan et al., 2009; Angelidis et al., 2018) and might allocate more regulation effort to the elevated arousal experienced during the ascending sound context. The low theta/beta ratio individual would be less sensitive to unattended stimuli changes. However, participants who were inferior in attentional control might be more susceptible to distraction, especially for those having higher theta/beta ratio in individuals diagnosed with ADHD or anxiety disorders (Derakshan et al., 2009; Arns et al., 2013). In unattended situations, they might be more sensitive to changes in terms of response speed. They might also show attenuated neural response, due to the non-linear relation between theta/beta ratio and HRV.

Summarizing, sensory stimuli do not reflect the simple pattern of immediate energy; rather they contain focal, contextual, and organic components (regulation) (Helson, 1964). The pooled effect of these components determines and maintains a neural trace of the contextual effect, where the focal component of a deviant stimulus impinges upon organisms already adapted to the context (Helson, 1964; Buonomano and Maass, 2009; Chambers et al., 2017). With different ambient auditory contexts, our data showed the dissociation of the presumed negative correlation between amplitude and latency of the MMN response. Perhaps contexts influence MMN on both physiological and psychological aspects. Latency of the MMN changes depends on the time when the deviance in stimulus is detected, thus to the threshold of the sensory system for the change, which is rather physiological and thus objective. The amplitude of the MMN, on the other hand, changes depending on the salience of the deviance for the subject, which is rather psychological and thus subjective. Therefore, assessing perception and adaptation to ambient environmental changes requires a more holistic perspective including context along with regulation capacities.

## Ethics Statement

This study was carried out in accordance with the recommendations of Cornell University Institutional Review Board with written informed consent from all subjects. All subjects gave written informed consent in accordance with the Declaration of Helsinki. The protocol was approved by the Institutional Review Board.

## Author Contributions

YH and DG proposed the study concept and designed the experiment. YH and QS collected the data. YH and LY analyzed the data. YH wrote the manuscript with contributions from co-authors.

### Conflict of Interest Statement

The authors declare that the research was conducted in the absence of any commercial or financial relationships that could be construed as a potential conflict of interest.
