# Supplementary material for: Interaction of Self-Regulation and Contextual Effects on Pre-attentive Auditory Processing: A Combined EEG/ECG Study
Source: Front Neurosci. 2019 Jun 19;13:638. doi: 10.3389/fnins.2019.00638 (PMC6593616; doi:10.3389/fnins.2019.00638)
Supplement: Supplementary file 1 [file Data_Sheet_1.PDF]

Supplementary Material

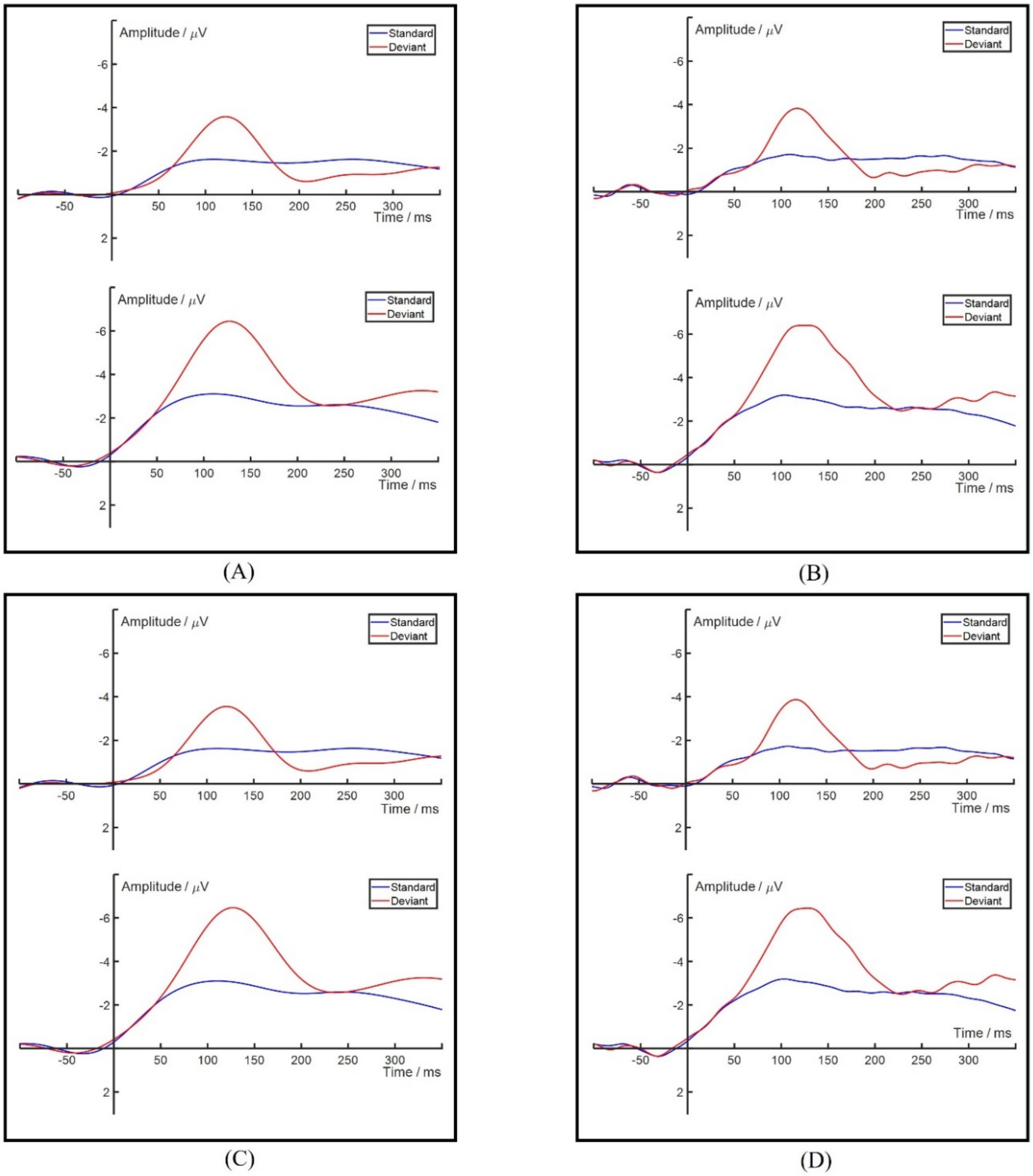

Figure S1. The influence of the filtering parameters on the ERP waveforms with respect to the descending and the ascending sequence. (A) The EEG signal was filtered within 0.5 and 10 Hz, with the upper one corresponding to the descending sequence and the lower one corresponding to the ascending sequence. (B) The EEG signal was filtered within 0.5 and 40 Hz. (C) The EEG signal was filtered within 0.1 and 10 Hz. (D) The EEG signal was filtered within 0.1 and 40 Hz.

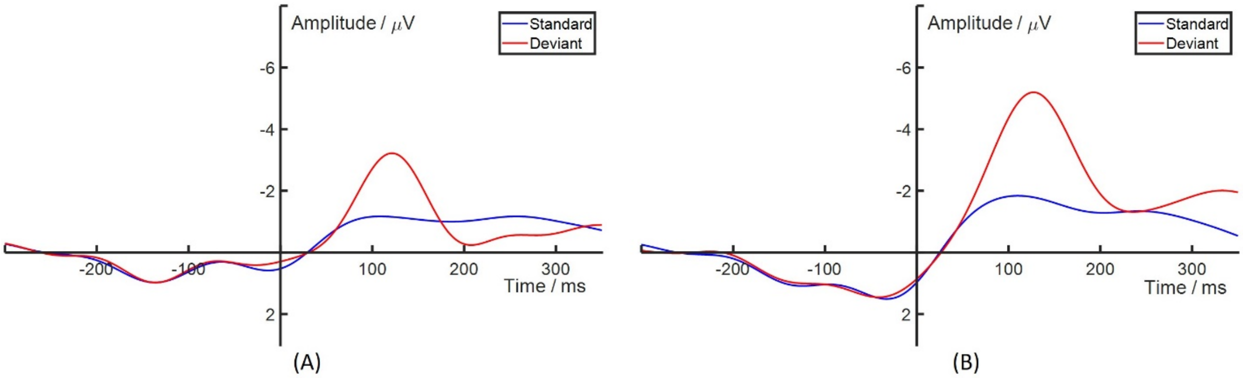

Figure S2. The ERP with respect to descending sequence in (A) and ascending sequence in (B). The baseline was within [-300 -200] ms, during which period no stimulus was presented.

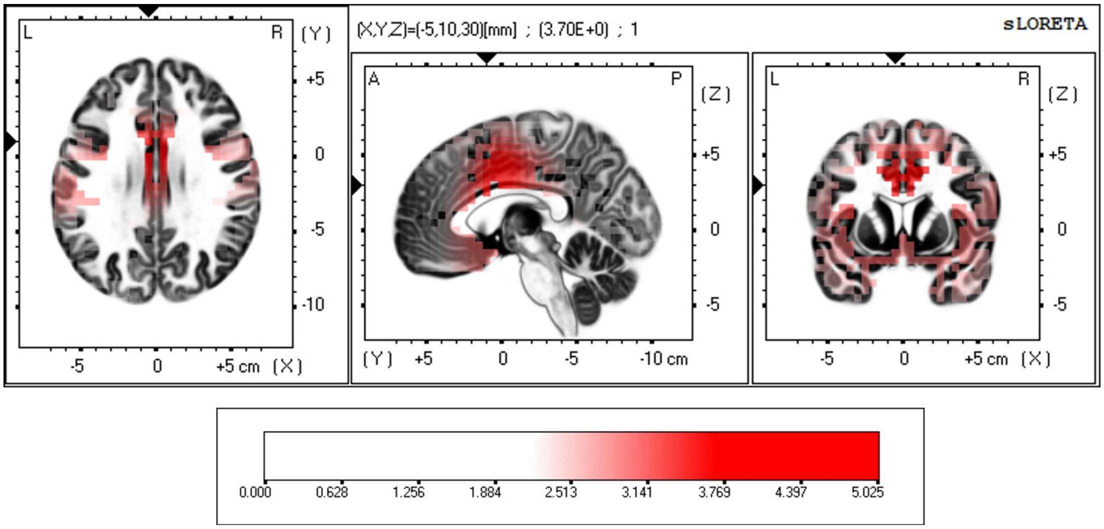

Figure S3. Statistics (*t* value) of the current source density of the MMNs (0.5~55 Hz) in the ascending condition compared with the descending condition.

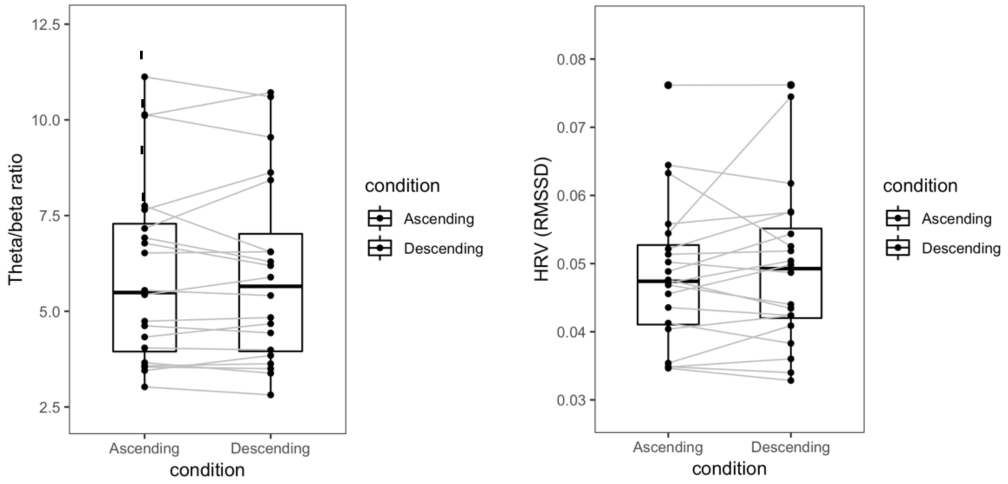

Figure S4. Theta/beta ratio and HRV in two conditions. Paired *t*-test, theta/beta ratio: *t* (19) = -0.77, *p* = 0.4485, RMSSD: *t* (19) = 0.08, *p* = 0.9360

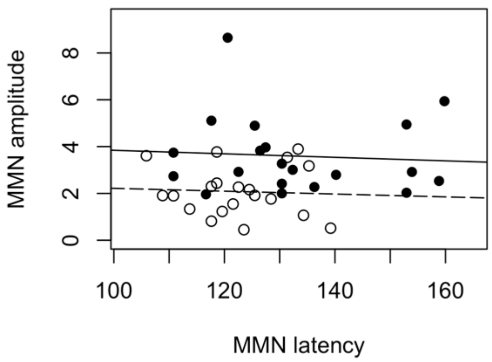

Figure S5. Correlation between MMN latency and amplitude. Across conditions, *r* = 0.14, *t* (38) = 0.83, *p* = 0.4097. In the ascending condition, *r* = -0.07 *t* (18) = -0.30, *p* = 0.7664; in the descending condition, *r* = -0.05 *t* (18) = -0.22, *p* = 0.8259.
